# What are the ethical limits of claimed scientific authorship? A case report of relevance

**DOI:** 10.1007/s00018-025-05650-8

**Published:** 2025-03-17

**Authors:** Juan-Carlos Argüelles

**Affiliations:** Área de Microbiología, Facultad de Biología, Universidad de Murcia, 30100 Murcia, Spain

**Keywords:** Metric parameters, Scholar repercussions, Ethics, Authorship, Autophagy

## Abstract

Since its discovery in the middle of the XX century, research into autophagy has undergone a spectacular expansion, particularly in the early 1990s. A number of physiological processes involving autophagy have been revealed and important human pathologies have been associated with perturbations in autophagy. In 2008 the “Guidelines for the use and interpretation of assays for monitoring autophagy” was launched with the purpose of collecting in a single document all the available information to monitor autophagy, which, it was thought, might be useful for established groups and any new scientists attracted by this field. The usefulness and success of this Guidelines has led to the subsequent publication of editions every 4 years, a task in which a growing number of authors have become involved and consequently included in the list of contributors. However, this worthy initiative and closely associated metric parameters has led to important scholarly repercussions in terms of perceived merits, grants and financial support obtained, professional careers and other areas concerning scientific activity. All these aspects are carefully examined in this contribution.

## Overview

The process of autophagy has been highly conserved throughout evolution, where it has been seen to play an essential role in the physiology of eukaryotic organisms as a major cellular degradation pathway [1]. This regulatory mechanism carries out the selective elimination of several cellular components including misfolded proteins, altered organelles and even intracellular infective pathogens. Although the existence of autophagy processes has been known since the 1960s, they only began to arouse great interest on the part of the scientific community at the beginning of the 1990s [1], since when intensive research effort by respected research groups has allowed crucial questions regarding the genetic and biochemical basis of autophagy to be elucidated.

As a reflection of the progress made and the worldwide impact of this topic, the 2016 Nobel Prize for Physiology or Medicine was awarded to the Japanese scientist, Yoshinori Oshumi “… for his discoveries made on the mechanisms involved in autophagy”. A large part of his decisive research took the budding yeast *Saccharomyces cerevisiae* as a biological model. Although a specialized journal exclusively devoted to this subject, *Autophagy*, was launched in 2005 and despite the impressive level of knowledge that has been achieved, some unanswered questions and challenges remain. Recently, such topics have been re-examined in a survey by a group of editors and specialists in distinct fields that involve the processes of autophagy [2].

In 2008 and in the academic format of a “review”, the journal *Autophagy* published the “Guidelines for the use and interpretation of assays for monitoring autophagy in higher eukaryotes” (Table 1) [3] (henceforth referred to as *Guidelines*). According to its presentation, this initiative, led by Dr. D.J. Klionsky, Editor-in-Chief of *Autophagy*, was taken following the acceleration of research in the field, and the need to establish essential criteria for demonstrating autophagy. The *Guidelines* pursued the laudable goal of providing in a single document a selection of the most appropriate methods for monitoring autophagy, which might be considered useful for new scientists entering the field. Furthermore, additional complementary information covering important aspects involving autophagy was also compiled [3]. From the beginning, the *Guidelines* had great success among research teams devoted to this area. Propelled by the exponential growth of new experimental data as well as by the exciting conceptual advances recorded, three further editions of the *Guidelines* have been published with an approximate periodicity of four years, in which the expression “in higher eukaryotes” was suppressed [4–6] (Table 1).

**Table 1:** Relevant bibliometric parameters appearing in successive editions of the “guidelines for the use and interpretation of assays for monitoring autophagy”. The ISI web of knowledge database is taken as reference

Edition	Year^a, b^	Authors	Citations^c^	Pages
1^st^	2008	236	1,995	25
2^nd^	2012	1,267	3,642	100
3^rd^	2016	2,460	5,030	223
4^th^	2021	2,981	1,595	383

^a^ The complete title of the 1^st^ edition (2008) is: “Guidelines for the use and interpretation of assays for monitoring autophagy in higher eukaryotes”

^b^ For each edition, the full reference has been included in the corresponding list (Klionsky et al. 2008, 2012, 2016, 2021)

^c^ The last access to the database was on 20 January 2025

It is undisputable that the *Guidelines* has had important repercussions, not only as regards the day-to-day work carried out by the scientific community, but also in social fields that go beyond specific research on autophagy. However, before dealing with my personal - and therefore subjective - analysis regarding the various editions of *Guidelines* and their consequences in different spheres, I think it is important to examine the scientific and mediatic context in which this initiative was adopted 15 years ago.

## Scientific research in the XX Century: changes in the paradigm and metric factors

### Impact of metric policies

The scientific revolution that coincided with the beginning of the XX century was the result of a decisive paradigmatic change. In all major fields of research, scientists, financially secure themselves or supported by rich patrons, were replaced by large multidisciplinary teams endowed with considerable human and material resources. Such teams usually adopted a hierarchical structure, headed by a leader or director, followed by senior, usually post-doctoral scientists and pre-doctoral students in different stages of their scientific formation. In addition, groups welcomed short-stay visitors or sought collaborators in topics of immediate interest. The universal value of science per se and its socio-economic implications that arose from this new paradigm, has turned scientific research into a demanding competitive task between groups, which must compete in open calls by presenting the “best” projects to ensure their financial support.

As a result, public and private institutions have laid down a set of apparently rigorous criteria designed to choose excellence over mediocrity, the representatives of the latter being condemned to rejection. Such criteria are based on a set of metric parameters presumed to act as “objective” filters of excellent research: quantity and quality of publications, citations, h-index, “category” of Journals expressed through their Impact factor (IF), first quartiles, deciles, etc. Their strict application gives rise to a mathematical result that will decide the success or failure of applications for projects, grants and/or appointments.

Nevertheless, many critics have looked carefully at the validity of this procedure and questioned its apparent impartiality [7–9]. Indeed, consistent evidence has demonstrated the pitfalls of this metric policy, such as short-term repetitive research, poor planning and trivial goals, an unjustifiable pressure on the teams for inconsistent and unrealistic publications and grant applications [8–10]. In fact, the use of IF in the case of both academic and scientific positions is based on unwarranted inferences [9, 10]. The temptation to follow such behaviour could well lead to fraud, misconduct [7], plagiarism or false authorship among other unethical manifestations contrary to the spirit of Science [7–10]. In this respect, the San Francisco Declaration on Research Assessment (DORA) strongly opposed the application of journals’ IF to evaluate the merits of scientists [11, 12]. However, despite these severe flaws and other problems, such policy is widely applied outside scientific areas, with serious consequences, not only of an ethical nature, but also academic and scholastic consequences for those researchers who are also engaged in teaching careers [13, 15] (see below).

### Case study: the four editions of autophagy guidelines

It is in the light of the above scenario that the true role played by the *Guidelines* must be analyzed in detail. Table 1 presents some relevant metric data which can be directly compared between the successive four editions [3–6]. It is not our purpose to question whether their inclusion in this analysis is or is not correct, an aspect that has previously been discussed and widely examined in several studies mentioned in the bibliographical references [7, 9, 11, 12]. However, it is certainly indisputable that several internationally homologated databases (among them the ISI Web of Knowledge and Scopus), when referring to the *Guidelines* assign the same number of citations to all listed authors on the understanding that each author’s individual contribution is equivalent to that of others. This crucial point cannot be underestimated.

At first sight, the figures are more eloquent than any argument concerning the importance and significance assigned to autophagy within scientific research. Of note is the 16-fold increase in the number of pages included in the *Guidelines* since its first publication 15 years ago. There has also been a significant increase in the total number of citations recorded (Table 1). On the other hand, a direct comparison between successive editions would not be justified in terms of the overall number of citations because the time elapsing since the date of publication would tend to favour those corresponding to the earliest editions rather than the most recent.

### Considerations on the number of authors

Perhaps the most attention-grabbing aspect is the increase in the number of the authors that have contributed to the successive editions of the *Guidelines* (Table 1), an aspect that deserves special attention because of the implications for the scientific prestige of the groups concerned and the academic standing of individual researchers: This is not to mention such important implications as media impact, the capacity to attract public and/or private funding, or the social relevance of research teams and those who form them [9, 11, 13, 14]. For example, assuming that all the signatories have contributed equally, we must bear in mind the enormous effort necessary to integrate in one manuscript the direct and inevitably varied work generated by 1,000 (2nd edition) or 2,000 (3rd and 4th editions) individual authors spread around the world.

On the other hand, regarding professional careers and scholarly reputations, it should be borne in mind that, as a rule, the permanent positions within a research team are few, limited to the main leaders and professors, who have only attained them after a long trajectory of meritorious work, personal dedication and well- earned success [13, 14]. But the financial rewards for initial predoctoral students and fellows tend to be poor and in the form of temporary, insecure contracts. Only a small percentage of initial candidates reach the final goal of getting an academic position as professor or researcher, and the metric results mentioned above are an essential factor for evaluating this complex scientific and life-long journey. As a consequence, all the researchers included as authors in the *Guidelines* together with the proper authorship are also rewarded with a high number of citations (more than 1,500 in the 4th ed.), giving them great advantage in their future careers and/or grant applications. From our personal viewpoint, it seems rather strange that among this plethora of authors, there is no mention of Y. Ohsumi, a Nobel Prize winner for his research on autophagy.

Nevertheless, it is important to remark that the *Guidelines* is not an exceptional case of a scientific publication signed by a vast number of authors. A similar concern about the actual contribution of listed authors may be applied to hundreds, if not thousands, of papers. However, the availability of serial instructions manuals is required in some important areas of medical research. This is the case of many clinical guides that need to be periodically updated to include new advances in basic knowledge of pathogenic organisms, diagnostic methods, therapeutical treatments, etc. In this way, the periodical edition of the *Guidelines* allows us to form a clear picture of the tremendous importance and new knowledge reached in the field of Autophagy in the last two decades [1–4].

### Benefits for the receiving journals

Competition is not confined to research scientists, as the journals themselves are immersed in an arduous battle to increase their prestige among the scientific community and publish the “best” articles. Editors therefore aim to reach the highest IF values and receive the most citations on the understanding that both parameters are closely linked to the quality and scientific level of the articles published [15–17]. Thus, the peer-review process becomes more demanding and editors will have a wider margin to act as inflexible and selective filters, choosing only those contributions that, in their judgment, represent a substantial advance in scientific knowledge [17–19]. It is also true that the journal itself and its publishers receive substantial benefits in terms of publicity. In the case of *Autophagy*, it is obvious that the large numbers of citations attained through the *Guidelines* improve its visibility, IF and ranking compared with other journals in the field. Thus, it is to be expected that groups in the field will tend to send it articles for evaluation. Meanwhile, the decision of the American Society of Microbiology (ASM) to withhold mention of IF from the web pages of all its journals does not seem to have had any effect to date [19–22]. In fact, panels of experts for grants concession make their decisions mainly based on bibliometric indicators, despite their drawbacks [18].

Such economic, academic and professional aspects associated with scientific research require a deeper analysis. The growing importance in the design of scientific policies played by publications and their associated metrics, together with commercial expectations, has led to an explosion of new journals in on-line format, in which editorial policies have been reversed. Open Access permits any content to be read free of charge, the authors themselves and agreement among institutions obviating the need for expensive subscriptions. In theory this strategy will lead to any article being accepted, as long as basic standards of quality are met, without the drawbacks associated with many journals that claim they receive many more contributions than they can publish. In reality, however, it has been detected that evaluations are frequently made by editorial boards, involving lax policies, a limited number of peer reviews, and few rejections, resulting in a clear drop in rigour and the evaluation of scientific publications of doubtful quality [23]. However, many journals under suspicion have reached a high metric index to the detriment of relevant classic journals, which have historically published seminal papers. These now remain as residual journals [24].

### Final considerations and perspectives

In principle, it is not easy to identify the researchers with sufficient merit to be considered as true authors of a publication. This is especially true in light of the comments mentioned above. One obvious criterion is that an author can only be considered if they have made a significant contribution to the research in question. However, several initiatives adopted by certain publishers in an attempt to establish and disclose the individual inputs to a submitted paper have turned out to be unsatisfactory [16, 18]. In this sense, metric parameters should be applied in combination with other criteria that better reflect the true authors’ contribution (i.e. number, position and corresponding role). Whatever the case, the number of authors “signing” many papers is hugely disproportionate (e.g. 2,981 in the 4th ed. of the *Guidelines*), and still worse if they are all considered intellectual “fathers of the creature”. This scenario has professional repercussions for those scientists who need to compete for grants or academic positions with other colleagues, whose publications might be over-rewarded by unfairly collected citations. Likewise, it is also unrealistic and incompatible with rigorous scientific procedure that the work of one investigator can give rise to more than 50 published papers per year. These and other flaws are unacceptable. According to E. Haeckel: “The scientific results of an institute are in inverse proportion to its size”.

In this context, some criteria on this polyhedral and complex matter may be recommended. (i) Firstly, a distinction must be made between individual researchers and multidisciplinary scientific teams. In this way, an enormous number of authors and/or a similar level of citations might be regarded as a possible case of misconduct, equivalent for instance to editorial frauds or so-termed “predatory” journals. (ii) As a general rule, a person should only be included as an author in a scientific manuscript when he has made a significant conceptual and/or experimental contribution to the matter under study. Sporadic consultations and suggestions, critical reading and other non-essential roles can be mentioned in the acknowledgements. Furthermore, the “obligatory” presence in the authorship of group leaders(s) who have merely provided inspiration for the research must also be questioned, even if their mention and prestige are important in the search for new grants and financial support. (iii) Although it is difficult to establish the maximum number of authors for a paper, some threshold limits must be agreed amongst researchers and publishers (a flexible range seems reasonable depending on the scientific field). (iv) It is true that some scientific breakthroughs require multidisciplinary collaboration from distinct groups with continuous interaction among specialists. In this way, when a consortium is constituted for projects in front-line research, any resulting publications must include all the participants. Perhaps, even, a new category of articles might be proposed, although this should be an exception rather than a rule. Alternatively, these publications may exclusively refer to the institutions participating in the study, but not the individual investigators. In fact, this strategy is already operative in some private companies. (v) Likewise, there should be a consensus concerning all clinical Guides and similar instruction manuals, so that they are no longer considered scientific articles *sensu stricto*. Compilations and/or methodological reviews might be the only format admitted as feasible. Therefore, they cannot receive any citation or any other metric index. Of course, as stated above, this rule does not question the scientific relevance of the contributions. (vi) Given that this is not an easy task, it should be established that both research teams and committees must take special care to identify objective rules and precise limits regarding the ethical and methodological requirements necessary to be considered as a true author of a scientific monograph.

A careful survey of the history of scientific research reveals how many prominent breakthroughs have involved a dare or objection from firmly accepted viewpoints. Many of them were ignored and/or rejected by the official establishment. This trend continues to the present day, when scientific research has changed its main goal from “discovery” to “production” [25]. This flawed policy tends to exclude those heterodox scientists considered as mediocre because of their low level of publications. However, it is common to find that most crucial scientific breakthroughs are not achieved by large research groups, but rather by courageous and outstanding individual personalities who challenged accepted and apparently solid paradigms by proposing audacious hypotheses. Inclusion in a list of authors, albeit many times, is not necessarily a reflection of excellence.

## Data Availability

N/A.
